# Biotechnological production of carotenoids by yeasts: an overview

**DOI:** 10.1186/1475-2859-13-12

**Published:** 2014-01-21

**Authors:** Luis Carlos Mata-Gómez, Julio César Montañez, Alejandro Méndez-Zavala, Cristóbal Noé Aguilar

**Affiliations:** 1 Food Science and Technology Program, School of Chemistry, Universidad Autónoma de Coahuila, Saltillo, Mexico; 2 Chemical Engineering Department, School of Chemistry, Universidad Autónoma de Coahuila, Saltillo, Mexico; 3 Group of Bioprocesses, Food Research Department, School of Chemistry, Universidad Autónoma de Coahuila, Saltillo, Mexico

**Keywords:** Pigments, Yeast, Agroindustrial wastes

## Abstract

Nowadays, carotenoids are valuable molecules in different industries such as chemical, pharmaceutical, poultry, food and cosmetics. These pigments not only can act as vitamin A precursors, but also they have coloring and antioxidant properties, which have attracted the attention of the industries and researchers. The carotenoid production through chemical synthesis or extraction from plants is limited by low yields that results in high production costs. This leads to research of microbial production of carotenoids, as an alternative that has shown better yields than other aforementioned. In addition, the microbial production of carotenoids could be a better option about costs, looking for alternatives like the use of low-cost substrates as agro-industrials wastes. Yeasts have demonstrated to be carotenoid producer showing an important growing capacity in several agro-industrial wastes producing high levels of carotenoids. Agro-industrial wastes provide carbon and nitrogen source necessary, and others elements to carry out the microbial metabolism diminishing the production costs and avoiding pollution from these agro-industrial wastes to the environmental. Herein, we discuss the general and applied concepts regarding yeasts carotenoid production and the factors influencing carotenogenesis using agro-industrial wastes as low-cost substrates.

## Introduction

Carotenoids belong to the most important components in foods. They are natural colorants, as yellow to red colors, so they have great influence on the acceptability of many foods. Moreover, some carotenoids are precursors of vitamin A; in terms of human health, they are among the bioactive phytochemicals credited that reduce risks for degenerative diseases such as cancer, cardiovascular diseases, macular degeneration and cataract [1]. Carotenoids are naturally occurring lipid-soluble pigments, the majority being C_40_ terpenoids, which act as membrane-protective antioxidants scavenging O_2_ and peroxyl radicals; their antioxidant ability is apparently attributed to their structure. Carotenoids pigments occur universally in photosynthetic systems of higher plants, algae and phototrophic bacteria. On the other hand, in non-photosynthetic organisms, carotenoids are important in protecting against photo-oxidative damage. Thus, many non-phototrophic bacteria and fungi rely on carotenoids for protection when growing on conditions where light and air are abundant [2].

Interest in carotenoids has increased considerably, mainly due to the benefits to human health and also to the growth of certain areas such as agriculture, especially aquaculture and poultry industry [3], Britton, nutritional supplements [4] food industry where they are used as coloring agents for cooked sausages, soft drinks, baked goods and pharmaceutical as additive to cosmetics [5,6]. Carotenoids market has resulted interesting in 2010 estimated at nearly $1.2 billion, but the expectations for 2018 are increasing considerably supposing to reach $1.4 billion with a compound annual growth rate of 2.3 [7].

### Classification and nomenclature

**Figure 1:**
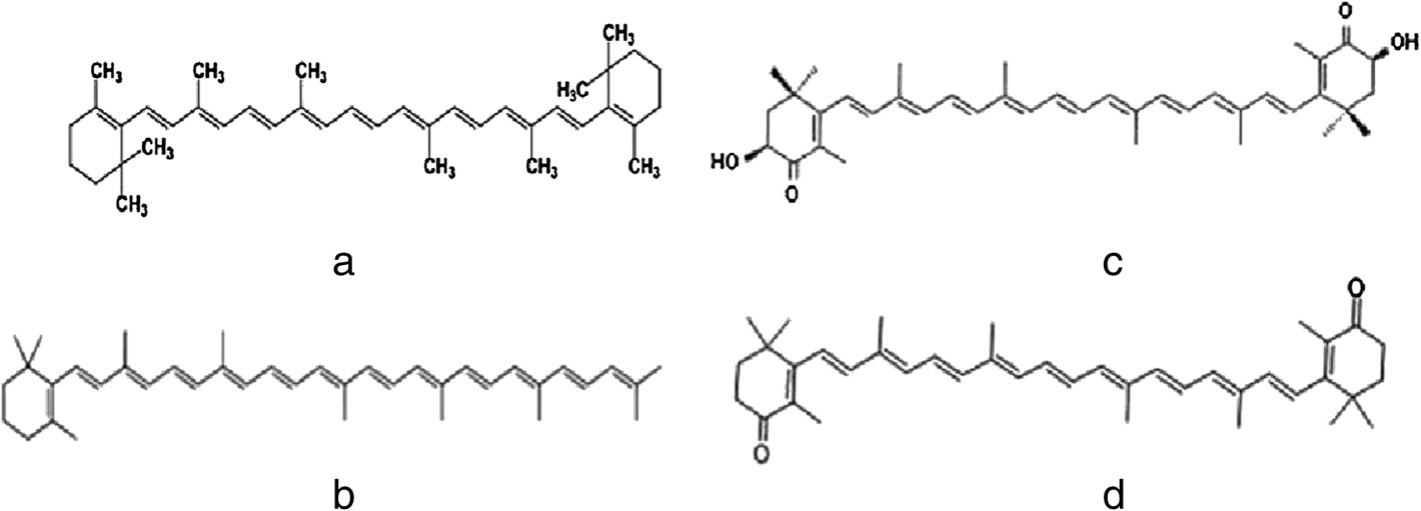
Molecules of carotenes: a) β -carotene, b) Torulene; xanthophylls: c) Astaxanthin, d) Canthaxanthin.

Carotenoids are terpenoid pigments of 40 carbon atoms derived biosynthetically from two units of geranyl-geranyl transferase pyrophosphate, they are soluble principally in nonpolar solvents. These pigments are grouped in carotenes and xanthophylls. Some carotenes only have carbon and hydrogen on their chemical structure such as β -carotene and Torulene; while xanthophylls also contain oxygen such as Astaxanthin and Canthaxanthin. Chemical structures of some carotenes are shown in Figure 1[8].

Generally, carotenoids are denominated according to structural variations of lateral rings, specially the double bond position. In general, carotene is used for the suffix carotene; and xanthophylls for the suffix ina [2].

Some of the microbial carotenoids produced by yeasts reported are:

•β -carotene (β-β’-carotene) (Figure 1a) this molecule has been used as food colorant or as a food supplement acting as provitamin A, in a concentration ranges from 2 to 50 ppm. It also is added to juices and drinks formulations (hydrophilic matrices) and others (lipophilic matrices) such as butter, margarine and cheese [9].

•Torulene (3′, 4′-didehydro –β, ψ-carotene) (Figure 1b) owns 13 conjugated double bonds, and has an attractive color. The antioxidant properties of torulene are attributed to its conjugated double bond system; in fact Torulene has more antioxidant efficiency than β -carotene, which presents less double bond on its chemical structure than torulene [10,11].

•Astaxanthin (3, 3′-dihydroxy-β,β -carotene -4,4′-dione) (Figure 1c) is a red pigment that causes coloration in marine invertebrates, fish and birds. Likewise carotenes described before it is applied as a colorant to confer the typical pink-red color of farmed salmon, trout and shrimp [12,13].

•Canthaxanthin (β-β′-carotene-4, 4′ -dione) is a cosmopolitan keto-carotene that is of interest by food and cosmetic industries, mainly [14].

### Biological properties of carotenoids

Commonly, carotenoids are distinguished as a vitamin A precursor, for this reason they are known as high-value nutritional molecules. The low intake of vitamin A is a nutritional problem in underdeveloped areas of the world. Its consequences are deficiency on tears production, blindness, principally in children, plus premature death [15]. Thus, vitamin A also shows other systemic functions maintaining like growth and reproductive efficiency, besides maintainings the epithelial tissue [9,16,17]. The carotenoid molecule provides antioxidant properties when it presents a cyclic structure joined to its carbons chain [18].

The antioxidant features of these compounds does not depends on its pro-vitamin A activity, because its capacity to attract oxygen with its double bond [17-20]. Apart of the properties described above, carotenoids also promote healthy effects including: improvement of immunity and diminishing in the risk of degenerative diseases such as cancer, heart diseases, cataract and macular degeneration [21-23]. Carotenoids intake, can prevent photoaging and sun burning on skin, but they must be ingested during weeks as to increase their levels in blood, and consequently increase protection [18].

Natural carotenoids have similar molecular structure than synthetics, but natural carotenoids differ on molecular structure providing major benefits to health. Industrial production of natural carotenoids can be carried out through to i) biotechnological processes using filamentous fungi, yeasts, bacteria or microalgae, and ii) solid-liquid extraction from plants. It has been reported that in the worldwide production of β -carotene only the 2% is from natural sources [24].

### Microbial carotenoid production

The commercial carotenoids are obtained by extraction from vegetables [25] and chemical synthesis [26]. However, in the case of production and marketing of several colorants from plants origin there are some problems regarding seasonal and geographic variability that cannot be controlled [27]. Own its part; the chemical synthesis generates hazardous wastes that can affect the environment. Unlike these traditional methods, the microbial production of carotenoids shows great interest and safety to use. Microbial production has the advantage to use low-cost substrates, resulting in lower costs of production. This explains the increasing interest in production of microbial carotenoids as substitutes for synthetic carotenoids used as colorants in food [3]. Thus, microbial synthesis offers a promising alternative for carotenoids production.

Carotenoids are widely distributed in microorganisms including bacteria, yeast, fungus and algae*.* Commercial production of microbial carotenoids is highly efficient because they can be easily managed during the processes [28].

Several reports in literature have described carotenoids production by fungus. Papaioannou and Liakopolou-Kyriakides [29], reported the use of *Blakeslea trispora* to produce β-carotene. Another mould, *Phycomyces blakesleeanus* is also known for its capacity to produces β-carotene at industrial scale, has been studied by Almeida and Cerda-Olmedo [30]. Studies carried out by Cerda-Olmedo [31] demonstrated that the most productivity strains of *Phycomyces b.* reach their maximum carotenogenic potential on systems without agitation; unlike to *B. trispora* strains. Also different authors have described the production of carotenoids by yeasts like *Rhodotorula* spp. This yeast, widely distributed in nature, can biosynthesize specific carotenoids such as β -carotene, torulene and torularhodin, in different proportions [32,33]. The production of carotenoids by genus *Rhodotorula* varies between species, and is affected by medium constituents and environmental conditions. The amount of carotenoids produced by this genus can be classified as low (less than 100 μg g^-1^), medium (101 to 505 μg g^-1^) and high (more than 500 μg g^-1^) as reported by many others [34-38]. Many studies of astaxanthin production have been published using the yeast *Xanthophyllomyces dendrorhous* developing reliable biotechnological processes. So, yeasts are reliable microorganisms to produce carotenoids. Bacteria have been reported as producers of cantaxanthin mainly, a carotenoid of interest in poultry, fishery, cosmetics, medicine, pharmaceuticals and food industries. Among the most studied carotenoids producers bacteria are *Corynebacterium michiganense, Micrococcus roseus, Brevibacterium* spp.*, Bradyrhizobium* spp., *Gordonia jacobaea* and *Dietzia natronolimnaea*[39]*.*

Microalgae belonging to Chlorophyceae group are the most recognized as producers of important commercial carotenoids, highlighting the genus of *Chlorella, Dunaliella,* and *Haematococcus.* These microalgae are able to produce lutein, β -carotene and astaxanthin [40]. Specifically the production of astaxanthin by *Haematococcus pluvialis* requires certain conditions during its cultivation, because it changes its structure during the growth cycle [41,42]. This means that the physical properties and nutrient requirements of the algae change during de culture process, altering the optimal conditions for growth and carotenoid production [43].

In general there is a vast of microorganisms that can biosynthesize carotenoids; however still lack of information is missing regarding strategies to maximize production at industrial scale. Nevertheless, although there are many strategies for stimulation of carotene biosynthetic pathways in yeasts, attention is still focused on unexplored habitats for selection of hyper-producing strains, which is the important step toward the design and optimization of biotechnological process for pigment formation [1,44]. Synthesis of important natural carotenoid by yeasts leads to consider them to industrial scale [45].

Also to knowledge of metabolic engineering tools to manipulate the biosynthetic pathway for carotenoid production by yeasts is very important. In 1964 Simpson *et al.*[46] described a possible biosynthetic pathway for carotenoid synthesis. Later, Goodwin [47] revised the general pathways for biosynthesis of carotenoid by yeasts and concluded that the carotenoid biosynthesis pathway involves three general steps:

1) Synthesis begins with conversion of acetyl CoA to 3-hidroxy-3-methyl glutaryl-CoA (HMG-CoA), catalyzed by HMG-CoA synthase. Then, HMG-CoA is converted in mevalonic acid (MVA), this is the first precursor of terpenoid biosynthetic pathway. MVA is phosphorylated by MVA kinase and decarboxylation; into isopentenyl pyrophosphate (IPP).

2) IPP is isomerized to dimethyllayl pyrophosphate (DMAPP) with the addition of three IPP molecules to DMAPP, catalyzed by prenyl transferase into geranyl geranyl pyrophosphate (GGPP). Condensation of two molecules of GGPP produces the phytoene (the first C_40_ carotene of the pathway); which is subsequently desaturated to form lycopene.

3) Many cyclic carotenoids are derived from lycopene, as β -carotene, γ -carotene, Torulene, Torularhodin and Astaxanthin when it undergoes many reactions.

As it has been mentioned β -carotene can be produced by many microorganisms. Focusing on yeasts of the genus *Rhodotorula,* there are some species such as *Rhodotorula glutinis, Rhodotorula minuta, Rhodotorula mucilaginosa, Rhodotorula acheniorum and Rhodotorula graminis* recognized as carotenoid producers. Also other yeast as *Sporobolomyces roseus, Sporobolomyces salmonicolor* and *Sporobolomyces patagonicus* are carotenoids producers. These yeasts are known as the most producers of β -carotene, therefore they are the most studied. Besides β -carotene these yeasts produces other carotenoids identified as Torulene, Torularodine, and γ -carotene [1,32,48,49].

The culture conditions, such as carbon and nitrogen sources influence the carotenoid profiles in yeasts as *R. glutinis*, the main carotenoid produced likely is β -carotene (about 25-43% of total carotenoids) and Torulene, the second largest (about 28-30% of total carotenoid) [16]*R. glutinis* has been used to produce β -carotene cultivated in glucose, whey, sugar cane (*Saccharum* spp.) molasses supplemented with yeast extract, and fermented radish (*Raphanus sativus*) brine [41].

Recently the worldwide production of β -carotene has increased due to the widely applications in different areas. β -carotene is a carotenoid known as the main source of pro-vitamin A, which is used in the treatment of medical diseases, and also used as food colorant among other applications. β -carotene as a high value compound showed a global market of $ 233 million dollars in 2010, and is expected to reach $ 309 million by 2018 with an annual growth rate of 3.6% [8]. Other carotenoid with high added value is astaxanthin; it is mainly produced by the yeast *Xanthophyllomyces denrorhous* (*Phaffia rhodozyma).* Astaxanthin is a powerful antioxidant that has attracted industrial interest for the application in aquaculture, chemical, pharmaceutical, and alimentary industries [50-55]. Astaxanthin is the third most important carotenoid economically after β -carotene and lutein. Astaxanthin market reached the 29% of total carotenoid sales with a global market size of $ 225 million dollars, estimating increase to $253 million by 2018, approximately [8].

### Factors influencing the production of carotenoid in yeasts

Recently, researchers have been interested on biotechnological processes to obtain high added value metabolites like carotenoids, due to the benefits that they offer to different industrial areas such as pharmaceutical, medical, and food and beverages. Therefore the production of microbial carotenoids at industrial scale must look for low-cost processes, high yields, and environmental friendly. However, the biotechnological synthesis of carotenoids is influenced by many factors involved in the processes that can affect the yields and operation costs. Some of these important factors to consider are:

•Carbon source is the most studied parameter to influence carotenogenesis. Metabolism of yeasts acts depending on the kind of carbon source in the medium. Glucose and other fermentable sugars are metabolized by the glycolytic pathway, and then an alcoholic fermentation, even with oxygen [14]. On the other hand, the carbon sources no fermentable as ethanol and succinate is carried out through acetyl-CoA oxidation to enter the citric acid cycle. Many authors have mentioned that carbon sources as ethanol provoke an increase in the pigments synthesis [12,56-58].

•Light is an important factor to considerate during the production of microbial carotenoids; hence, it improves carotenogenesis [59]. Microorganisms need to prevent themselves from the light that causes damage, and carotenogenesis is a photoprotective mechanism [60-62]. Carotenoid production is affected positively by white light, and carotenoid concentration depends of the microorganism. In addition, carotenoid production is associated to the increasing of the enzymes activity involved in carotenoid biosynthesis [63]. Moliné *et al.*[49] studied the relationship between carotenoids and ergosterol and cell UV-light resistance in strains of *R. mucilaginosa*, and reported that the hyper-pigmented strains showed enhanced survival (250%). They also indicated that higher production of torularhodin improves UV-light survival in yeasts. On the other hand, Yen and Zhang [64] evaluated the β-carotene productivity in a batch reactor with two LED (light emitting diode) lamps resulting in a β-carotene concentration 24.6 μg g^-1^; whereas without light the β-carotene concentration was found to be 14.69 μg g^-1^.

•Temperature is another parameter to take into account in carotenoid production by yeasts, it affects the cell growing and metabolite production, it acts changing the biosynthetic pathways, including the carotenogenesis. The effect of temperature depends on the microorganism and the quantity of product [56,65-69]. Malisorn and Suntornsuk [70] optimized the temperature effect over carotenoid and biomass production in *Rhodotorula glutinis*, reporting at 29 and 30°C the maximum production, respectively. According to Hayman *et al.*[71], temperature effect regulates the enzyme concentration involved in the carotenoid production, and therefore the carotenoid levels in microorganisms are controlled.

•Aeration is another important parameter, due to carotenogenesis is an aerobic process, and the airflow rate in the yeast culture is an essential factor to the substrate assimilation for the growth rate, cell mass and carotenogenesis. Also, decreasing oxygen levels influences the production of carotenes or xantophylls, due to oxidation of carotenes in Astaxanthin, Cantaxanthin and others [12]. The effect of aeration depends on the species of the microorganism [72-87]. Saenge *et al.*[44] examined the effects of aeration rate on cell growth, lipid yield, carotenoids production and glycerol consumption. It was noticed that the aeration rate had a significant effect on biomass and lipids production, when it was increased from 0 to 2 vvm, the biomass and lipid yield were the highest at 8.17 and 4.32 g L^-1^, respectively.

•Metal ions and salts (Ba, Fe, Mg, Ca, Zn and Co) have also been demonstrated to be stimulants carotenoids production by *R. glutinis*[88]. Otherwise, Buzzini *et al.*[89] reported that certain trace elements have shown a selective influence on the carotenoid profile in *R. graminis*. In the case of Al^3+^ and Zn^2+^ a stimulatory effect on β -carotene and γ- carotene production was observed, while Zn^2+^ and Mn^2+^ showed inhibitory effect on torulene and torularhodin production. The effect of trace elements mentioned is attributed to the activation of specific carotenogenic enzymes [71].

•The solvents and chemical or natural agents have been studied regarding their effect on carotenogenesis. Kim *et al.*[90] reported that cell mass and astaxanthin production is stimulated by the addition of ethanol (10 g L^-1^) and acetic acid (5 g L^-1^) to a fed-batch culture of *P. rhodozyma* that resulted 45.62 mg L^-1^ and 43.87 mg L^-1^ of carotenoids concentration, respectively. Similarly, Gu *et al.*[58] reported increased carotenoid production (from 1.65 mg carotenoids g^-1^ cells to 2.65 mg carotenoids g^-1^ cells) upon addition of 0.2% (v/v) ethanol to cultures of the yeast *X. dendrorhus.*

The culture conditions, such as carbon and nitrogen sources influence the carotenoid profiles in yeasts as *R. glutinis*, the main carotenoid produced likely is β -carotene (about 25-43% of total carotenoids) and Torulene, the second largest (about 28-30% of total carotenoid) [16]*R. glutinis* has been used to produce β -carotene cultivated in glucose, whey, sugar cane (*Saccharum* spp.) molasses supplemented with yeast extract, and fermented radish (*Raphanus sativus*) brine [46]. An alternative to decrease the industrial operation costs during the synthesis of microbial carotenoids is the use of agro-industrial wastes as substrates.

### Low-cost substrates

Carotenoid pigments synthesis in yeasts starts at the late logarithmic phase continuing in the stationary phase [82,91,92], the presence of a reliable carbon source is important for carotenoid biosynthesis during the stationary phase. Yeasts can synthesize carotenoids when cultivated in commercial medium, containing various refined carbon sources, such as glucose [34,66,70,74,79,86,87,92-96], xylose [97], cellobiose [96], sucrose [98,99], glycerol [79] and sorbitol [91,96], nevertheless these type of medium represents high costs. As a result, studies on carotenogenesis have led to find out low-cost substrate as alternative to reduce production costs. Therefore, there have been a growing interest in the use of natural substrates as carbon sources such as grape juice [65,100]; grape must [101,102]; peat extract and peat hydrolyzate [65,82,103,104]; date juice of *Yucca filifera*[81], hydrolyzed mustard waste isolates [105], hemicellulosic hydrolyzates of eucalyptus globules wood [106,107], hydrolyzed mung bean waste flour [107], sugar cane juice [80,108,109], sugar cane and sugar-beet molasses [42,67,110], corn syrup [45,77], corn hydrolyzate [111,112], milk whey [67,71,72,75,78]. These by-products from industrial processes are pollutants to the environmental and their treatment represents high costs. In recent years, raw materials and agro-industrial wastes origin have been proposed as low-cost alternative carbohydrate sources. On this regard, chicken feathers and sweet potato have been introduced as nitrogen and carbon sources [113], respectively to produce carotenoid, and also minimizing environmental and energetic problems related to their disposal [114]. Many investigations have been performed to diminish the costs and optimize the carotenoid production; and factors such as carbon and nitrogen source are very important to consider on the selection of agro-industrial waste as substrates [115].

A wide spread natural substrate is milk whey; it contains lactose, proteins and minerals, principally. Biological wastewater treatment technologies can assist in safe disposal of whey within environmental specifications, but these are expensive [116] becoming an attractive low-cost substrate for microbial production of carotenoids. Carotenoid production by lactose-negative yeasts in whey ultrafiltrate could be carried out by enzymatic hydrolysis of lactose to single sugars (glucose and galactose), thus providing the method of co-cultivation with lactose-positive yeasts, producers of β-galactosidase [78], or modifying culture conditions under which lactose is hydrolyzed into carbon sources easily assimilated by the yeast [71,72,75]. Nasrabadi and Razavi [117] reported a carotenoid production of a lactose positive mutant strain (*R. acheniorum*), finding a yield of 262.12 ± 1.01 mg L^-1^ of β -carotene.

Aksu and Eren [68] reported that the addition of cottonseed oil to the *R. mucilaginosa* culture medium increased the production of total carotenoids with yield of 57.6 mg L^-1^ of carotenoids, while a concentration of 39.5 mg L^-1^ was reached without the addition of activators. Table 1 presents the most recent agro-industrial residues used as substrate to microbial carotenoid production, and the carotenoid quantities obtained. Production of carotenoid with agro-industrial wastes depends on the kind carbon and nitrogen source, minerals and other components, as well as the quantities of each one. The composition of these materials is very important to define the preparation of culture media to improve the carotenogenesis on microorganisms.

**Table 1:** Recent researches of use of agro-industrial wastes as substrates to yeasts carotenoid production

**Authors**	**Substrate**	**Yeast**	**Yield**
**Marova *et al.,*** [116]	Whey	*R. glutinis*	46 mg L^-1^ of β -carotene
**Marova *et al.,*** [116]	Potato medium	*R. mucilaginosa*	56 mg L^-1^ of β -carotene
**Saenge *et al.,*** [44]	Crude glycerol	*R. glutinis*	135.2 mg L^-1^ of carotenoids
**Taskin *et al.,*** [118]	Chicken feathers	*R. glutinis*	92 mg L^-1^ of carotenoids
**Nasrabadi and Razavi,**[115]	Whey ultrafiltrate	*R. acheniorum*	262 mg L^-1^ β -carotene
**Valduga *et al.,*** [119]	Whey	*S. salmonicolor*	590.4 μg L^-1^ of carotenoids
**Malisorn and Suntornsuk**[120]	Fermented radish brine	*R. glutinis*	19 μg L^-1^ h^-1^ of β -carotene
**Malisorn and Suntornsuk**[70]	Fermented radish brine	*R. glutinis*	201 μg L^-1^ of β -carotene
**Tinoi *et al.,*** [32]	Mung bean waste flour and sweet potato extract	*R. glutinis*	3.48 mg L^-1^ of carotenoids
**Frengova *et al.,*** [72,73]	Ultrafiltrate whey	*R. glutinis*	10.2 mg L^-1^ of carotenoids

### Biotechnological processes

Nowadays, the importance of biotechnological processes has increased due to benefits that they provide such as high yields, low costs and less waste disposals. These benefits are dependent on nutrients and the culture conditions such as inoculum size, pH, aeration, agitation, and others.

To design a biotechnological process, many factors must be taken in consideration such as bioreactor design, raw materials, the microorganism or enzyme, type of fermentation (batch, feed-batch or continuous). These features play a very important role to achieve the desired yields of a target metabolite [121]. The configuration and volume of the bioreactor is an important factor to consider in the microbial production of carotenoids. Several investigations using yeast for the production of microbial carotenoids in stirred tank bioreactors have been published (Table 2). A fully functional bioreactor offers advantages such as perfect integration of several components, ensuring that cultures will reach the desired productivity of microbial pigments or other microbial compounds, through an efficient and rigorous control of some parameters such as temperature, agitation, aeration, pH and dissolved oxygen among others.

**Table 2:** Bioreactors employed to carotenoid production with yeasts

**Authors**	**Bioreactor**	**Yeast**	**Yield (carotenoid)**
**Malisorn and Suntornsuk**[70]	3 L stirred tank	*R. glutinis*	201 μg L^-1^ (24 h)
**Ungureanu *et al.,*** [118]	3.7 L stirred tank	*R. rubra*	710 μg L^-1^
**Park *et al.,*** [122]	5 L stirred tank	*R. glutinis*	266.1 μg g^-1^ of biomass
**Luna Flores *et al.,*** [43]	3 L stirred tank	*X. dendrorhus*	8798.6 μg g^-1^ of biomass

**Figure 2:**
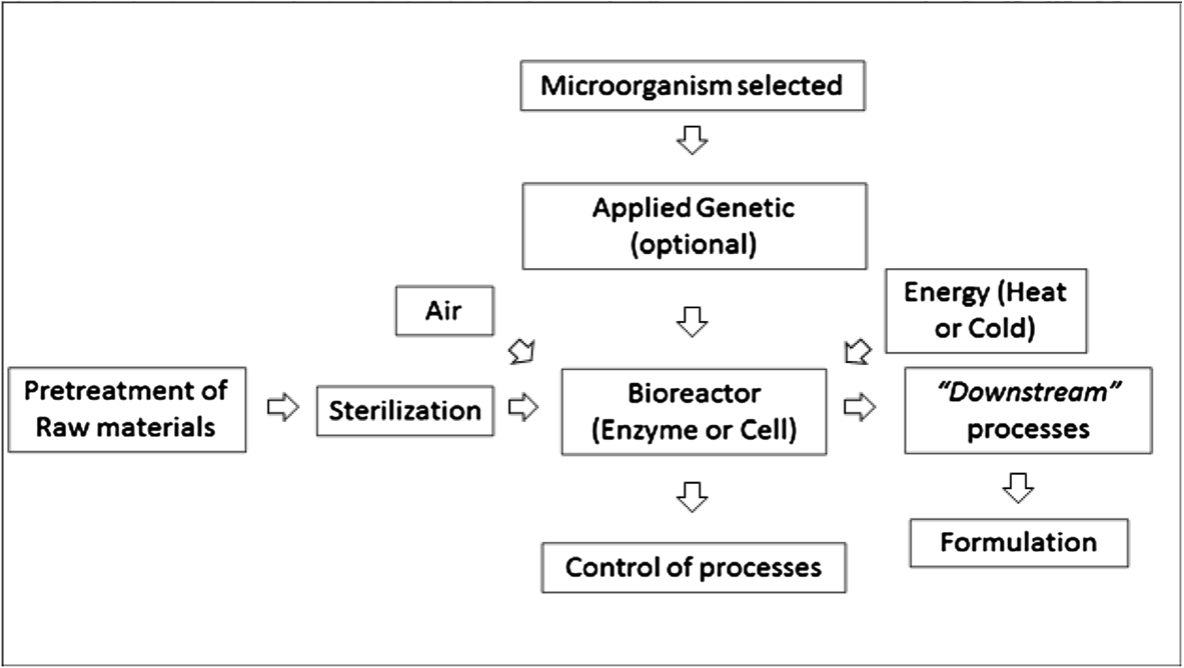
General scheme of biotechnological processes for carotenoids production.

Figure 2 illustrates a scheme of a general biotechnological process where the bioreactor is the central part of the process.

Industrial applications of bioprocess to produce microbial carotenoids generally employ genetically modified strains, being hyper-producing strains that had been manipulated to maximize carotenoids production. Among the several strategies to increase overall yield in a microbial carotenoids bioprocess, metabolic engineering for the yeast carotenoids producers in an interesting research area.

The microbial production of carotenoids involved several steps:

i) ***Selection of the appropriate substrate.*** The raw materials utilized might or not be pretreated depending on the fermentative capacity of the microorganism and the type of enzymes produced.

ii) ***Bioreactor.*** The bioreactor configuration and operational variables are crucial for the maximum yields of the process.

iii) ***Downstream processing.*** Production of intracellular pigments is limited by the complexity of “*downstream”* processing [123]. Cell disruption is a critical step to recover intracellular compounds and it affects recovery yield and carotenoids properties [124,125].

Carotenoid produced by yeasts as *Rhodotorula spp.* and *Phaffia rhodozyma* are synthesized and remained into the cells, therefore extraction processes are needed to recover the product of interest resulting in high costs of production [119]. The developing of recovery techniques of carotenoids has been an issue of study. Some techniques used for carotenoid extraction are shown in Table 3. These techniques used for cell disruption allow obtain the carotenoid formed into the cells. Actually, the downstream processing is the major challenge to recover intracellular pigments; many investigations have been performed to concerning the recovery of products; an ideal recovery process must be in non-time-consuming, low cost and high yields.

**Table 3:** Extraction of carotenoids using different techniques of cell disruption

**Authors**	**Disruption technique**	**Yeast**	**Yield (carotenoid)**
**Michelon *et al.,*** [123]	Freezing and maceration with diatomaceous earth	*P. rhodozyma*	93.13 μg g^-1^ of biomass
	Freezing and DMSO	*P. rhodozyma*	155.72 μg g^-1^ of biomass
	Enzymatic lysis and ultrasonic waves	*P. rhodozyma*	163.12 μg g^-1^ of biomass
**Aksu and Eren**[38]	Bead grinder	*R. glutinis*	125 mg L^-1^ of medium
**Park *et al.,*** [122]	Freeze-dried	*R. glutinis*	266.1 μg g^-1^ of biomass
**Valduga *et al.,*** [119]	Freezing in liquid N_2_ and maceration	*S. salmonicolor*	590.4 μg L^-1^ of medium
**Taskin *et al.,*** [118]	DMSO, acetone and petroleum ether	*R. glutinis*	92 mg L^-1^ of medium
**Gu *et al.,*** [58]	HCl and acetone	*Rhodotobacter sphaeroides*	4790 μg g^-1^ of biomass
**Buzzini *et al.,*** [78]	DMSO, acetone and petroleum ether	*Rhodotorula graminis*	803.2 μg g^-1^ of biomass

The development of biotechnological processes to produce carotenoid has recently increased because it is a reliable method to obtain carotenoids. Several patents related to microbial production of carotenoids have been registered worldwide (Table 4).

**Table 4:** Current patents of microbial carotenoid production

**Inventor**	**Title**	**Country**	**Year**
**Bailey *et al.,*** [126]	Carotenoid production in oleaginous yeasts and molds	MX	2012
**Mohamed *et al.,*** [127]	Biological production of Zeaxanthin and carotenoid biosynthesis control	US	2013
**Martin *et al.,*** [128]	Efficient astaxanthin production strains derived from *Haematococcus pluvalis*	US	2013
**Ruiz *et al.,*** [129]	Novel strain of *Lactobacillus plantarum* for the production of carotenoids	GRM	2010
**Yu *et al.,*** [130]	Method for preparing and extracting carotenoid from microbial thalli	US	2012
**Nanjun-Daswamy.,**[131]	Fermentation process to produce natural carotenoids and carotenoid-enriched feed products.	US	2012
**Bailey R *et al.,*** [132]	Production of carotenoid in oleaginous yeast and fungi	US	2010

### Metabolic engineering to production of carotenoids in yeasts

A strategy to reduce the costs of production is the obtaining of hyper-producer strains. Efficient techniques have been described to obtaining of mutant strains [120]. A simple technique is the random and selection mutagenesis through the color. For this techniques have been used chemical (Ethyl methane sulfonate, 1-methyl-3-nitroguanidine or antymicine A) and physical methods (UV light, gamma radiation).

Metabolic engineering is the improvement of cellular properties through the modification of specific biochemical reactions or the introduction of new ones, with the use of recombinant DNA technology [122]. Nowadays, the engineering in non-carotenogenic microorganisms for carotenoid production is a very useful tool. Metabolic engineering of microorganisms to produce higher levels of important molecules is a great possibility to improve public health through biotechnology [133]. The selection of appropriate microorganism is the first step in biotechnological processes, which could be undergone to mutagenesis to improve the strains and subsequently the production of metabolites [134].

Many authors have reported the application of metabolic engineering in yeasts, such as *Sacharomyces cerevisiae*[135-139] and *Candida utilis*[140,141]. The yeasts afore mentioned were modified successfully to production of carotenoids as: β -carotene, lycopene or Astaxanthin by the insertion of carotenogenic genes from *Erwinia uredovora, Agrobacterium aurantiacum* or *Xanthophyllomyces dendrorhus.* These yeasts are very useful in food industries, they are considered generally recognized as safe organisms by FDA (US) [142]. Bacteria such as *Escherichia coli*[143] have been described as microorganisms engineered to produce carotenoid, however *S. cerevisiae* is considered as a safe yeast and presents many advantages as easy genetic manipulation with established host-vector systems. Although naturally the yeast *S. cerevisiae* does not produce carotenoids, but it produces geranylgeranyl diphosphate, and if this yeast is integrated with the two main carotenogenic genes, phytoene synthase (*crtYB*) and phyotene desaturase (*ctrI*), from *Xanthophyllomyces dendrorhous* could produce carotenoids [144]. Being a characterized its genetic system, physiology and regulatory networks [133], this yeast is appropriate to be engineered to produce carotenoids.

An early attempt to produce β -carotene in *S. cerevisiae* was performed by Yamano *et al.*[145] using bacterial genes. The researching was successful in engineering the yeast to obtain β -carotene; nevertheless the level produced was low (103 μg/g dry wt.). Verwaal *et al.,*[140] studied the expression of carotenogenic genes which encode a bifunctional phytoene synthase and lycopene synthase from *X. dendrorhus*. The concentration of β -carotene obtained was 5.9 mg g^-1^ (dry wt.), with the improved strain. Also to produce β -carotene *S. cerevisiae* has been engineered for production of other carotenoids. Ukibe *et al.*[139] used genes encode to phytoene synthase (CrtI) and bifunctional phytoene synthase/lycopene cylase (CrtYB) from *X. dendrorhus.*

On the other hand, *Pichia pastoris* is other non-carotenogenic yeast that has also been studied to production of carotenoids, and it is able to grow in organic materials. Araya-Garay *et al.*[141] designed and constructed two plasmids containing the genes encoding lycopene and β -carotene. The results showed that obtained a recombinant strain, producer of lycopene and β -carotene reaching 1.141 μg and 339 μg per gram of dry biomass, respectively.

## Conclusions

Carotenoids are important molecules that improve foods quality due to their high nutritional value. For these reasons, carotenoid application as colorants has increased the interest of industries and scientists to develop low-cost process for carotenoid production. Biotechnological production mainly provides economics advantages over synthetic or extracted plant carotenoids. Yeast carotenoids have advantages such a high growth rate, decreasing the production time at industrial scale. On the other hand the use of low-cost substrates as agro-industrial wastes diminish the processes costs, and giving an alternative to the use of these wastes contributing to reduce environmental contamination. More research focused on the use of agro-industrial wastes, high carotenoids-producing yeast through metabolic engineering strategies, bioreactor design and control strategies for microbial carotenoids production are necessary to cover the worldwide demand by poultry, cosmetic, food, feed, beverages and others industries.

This review aims to show an overview on the biotechnological processes for carotenoid production, and the possibility to produce them at low costs, high yields and to reduce processing time. The knowledge about these processes is critical to improve and create new technologies in the production of microbial carotenoids.

## Competing interests

Authors declare that they have no competing interests.

## Authors’ contributions

LCMG contributed with the paper writing, data researching and the changes requested by the referees. AMZ supported the paper writing, data researching and revised the changes made to this paper. JCMS and CNAG revised and approved the changes made, and also with data researching and formatted the review. All authors read and approved the final manuscript.
